# Human oestriasis acquired in Florence and review on human myiasis in Italy

**DOI:** 10.1007/s00436-014-3906-9

**Published:** 2014-05-07

**Authors:** Lorenzo Zammarchi, Andrea Giorni, Simona Gabrielli, Marianne Strohmeyer, Gabriella Cancrini, Alessandro Bartoloni

**Affiliations:** 1 Clinica Malattie Infettive, Dipartimento di Medicina Sperimentale e Clinica, Università Degli Studi di Firenze, Largo Brambilla 3, 50134 Florence, Italy; 2 SOD Oculistica, Azienda Ospedaliera Universitaria Careggi, Florence, Italy; 3 Dipartimento di Sanità Pubblica e Malattie Infettive, Università “Sapienza”, Rome, Italy

**Keywords:** Myiasis, Italy, Ophthalmology, *Oestrus ovis*

## Abstract

Myiases-causing flies are widely observed in tropical countries, whereas in Italy, a temperate country, their epidemiology and clinical presentation are poorly known. We report three cases of human conjunctival myiasis recently observed at our hospital, and the results of a review of the literature on human myiasis in Italy. In August 2012, a case of *Oestrus ovis* conjunctival myiasis acquired in the city centre of Florence, Italy was diagnosed at our hospital. In the early fall of 2013, two additional cases, acquired in the neighbouring areas, occurred. The review of literature showed that, up to the middle of 1990s, myiasis in Italy was mainly an occupational disease of shepherds, caused by *O. ovis.* Recently, cases of travel acquired furuncular myiasis emerged, together with “opportunistic” autochthonous cases of wound myiasis in patients with underlying health conditions. Considering the causative agents of human of myiasis in Italy, among the 703 autochthonous cases reported, 98.1 % were caused by *O. ovis*, while among the 42 imported cases described, 59.5 % were due to *Cordylobia* spp. and 40.5 % to *Dermatobia hominis*. Our findings suggest that *O. ovis* conjunctival myiasis may still be observed in urban setting in Italy. Health care providers should know and implement the basic rules of entomoprophylaxis for myiasis in the facilities where they are working and use these indications to educate patients and care givers in both pretravel care and geriatric outpatient settings.

**Electronic supplementary material:**

The online version of this article (doi:10.1007/s00436-014-3906-9) contains supplementary material, which is available to authorized users.

## Introduction

Myiasis is defined as an infestation of living vertebrate animals, included humans, by fly larvae (Diptera, Brachycera, Cyclorrhapha) that develop until the L3 stage feeding on the host’s dead or living tissue or liquid body substances (Zumpt 1965). Clinical presentation of myiasis varies according to the causative species involved and to the anatomical location of the larvae (Francesconi and Lupi 2012). Each fly species has specific geographical distribution, but, in general, all species have a greater abundance in tropical countries, where the climate favours faster life cycles and a higher number of generations per year (Francesconi and Lupi 2012). In Italy, most autochthonous cases of human myiasis are caused by *Oestrus ovis*, still highly widespread in Italian flocks (Scala et al. 2001). We report three cases of autochthonous *O. ovis* conjunctival ophthalmomyiasis acquired in Florence area, Italy, by patients without history of any contact with environments particularly frequented by animals. Published literature on human myiasis in Italy was reviewed.

## Results

### Cases of *O. ovis* myiasis in Florence

**Fig. 1:**
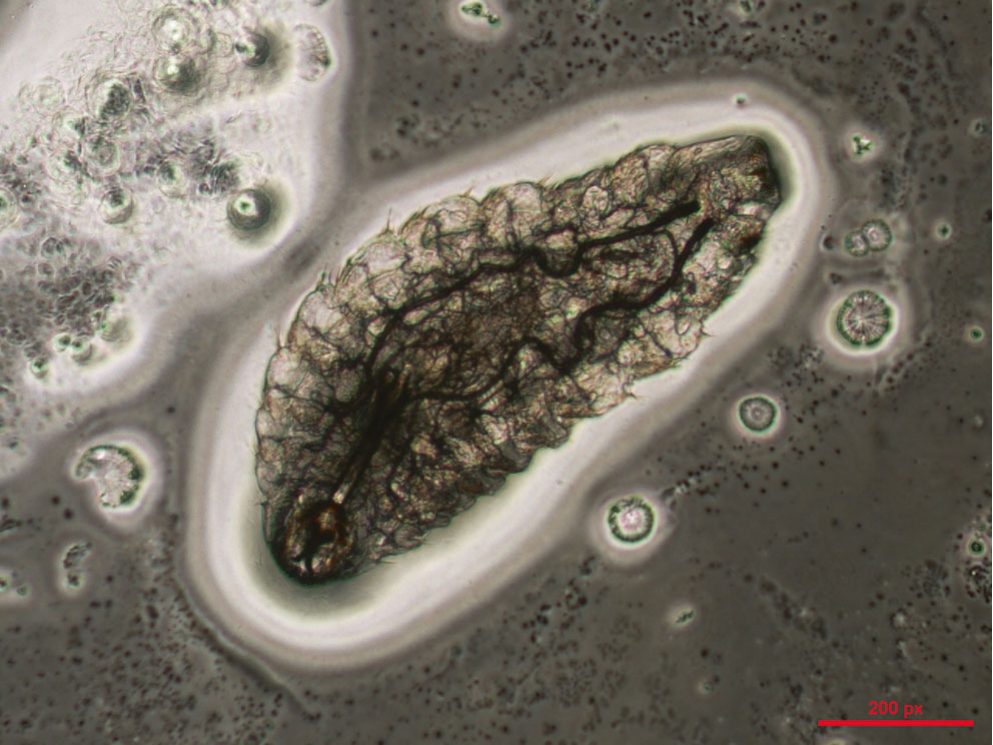
One of the first instar *Oestrus ovis* larvae recovered from the conjunctiva of the left eye of the patient observed in 2012

**Fig. 2:**
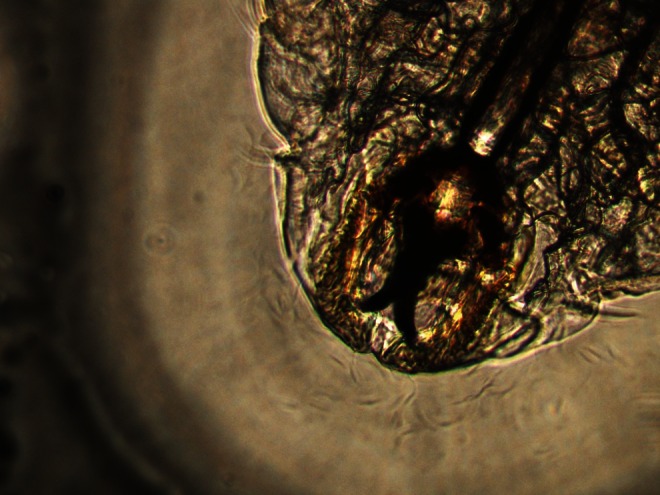
“Bull horns”-like buccal hooks in the cephalic extremity of the first instar *Oestrus ovis* larva recovered from the conjunctiva of the left eyes of the patient observed in 2012

**Fig. 3:**
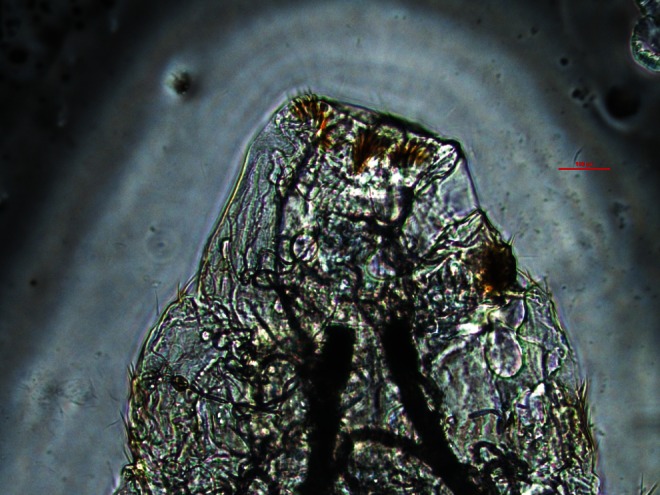
Typical spines in the caudal extremity of the first instar *Oestrus ovis* larva recovered from the conjunctiva of the left eyes of the patient observed in 2012

In August 2012, a 44-year-old woman was admitted to the ophthalmic emergency department of the Careggi University Hospital, Florence (Italy), complaining left ocular foreign body sensation, photophobia and lacrimation associated with rhinorrhea and sneezing fits. Symptoms had suddenly presented in the late afternoon of the same day and gradually worsened while she was walking in a public garden, near the city centre of Florence. Few minutes before the onset of symptoms, she noted the presence of an insect that was slowly flying around her. She had a visual acuity of 20/20 with spectacle correction and, on slit-lamp examination, there was a conjunctival hyperemia in the left eye with follicular reaction and mild eyelids oedema. Moderate superficial punctate keratopathy was present. Right eye was normal. Multiple cigar-shaped 1-mm long, clear-white, rapidly moving foreign bodies, showing tendency to avoid bright light, were found in both the inferior and superior left conjunctival fornices. The mobile bodies were presumptively identified as insect larvae by the ophthalmologist. After instillation of benoxinate 0.4 % eye drops, all the larvae were removed using forceps and abundant and repeated washing with normal saline and 5 % iodopovidone performed. Finally, the lacrimal ducts were flushed with saline. The removal was not easy due to the activity of the larvae; however, at the end of the procedure, ocular symptoms were completely resolved. Four larvae were stored in formalin and five in normal saline, whereas the remaining eight were washed away. The patient was discharged and prescribed with topical moxifloxacin 0.5 % four times a day, hydroxyzine dihydrochloride 25 mg and nasal douches with saline for 1 week, due to the persistent rhinorrhea and sneezing fits. Ophthalmologic and otorhinolaryngological follow-ups were scheduled, but no other larvae were found at repeated slit-lamp examination of the conjunctiva and at fiber-optic examination of nasopharyngeal cavities. Rhinorrhea and sneezing resolved in about 3 weeks after the larvae removal. Maggots were microscopically identified as *O. ovis* at first larval stage, given the observation of the “bull horns”-like buccal hooks in the cephalic extremity and the spines in the caudal extremity (Figs. 1, 2 and 3) (Guitton and Dorchies 1993; Rodhain and Perez 1985).

Two additional cases of conjunctival myiasis (involving the left eye in both cases) were observed at the same emergency department in September and October 2013, respectively. The first patient was an 84-year-old woman who lived in the country side in the Eastern Florence (Donnini), and the second one was a 28-year-old man who lived in an urban area near Florence (Sesto Fiorentino). Symptoms abruptly started in both patients while they were doing outdoor activities near their home. Both patients denied any attendance to grazing land or farms, both had clinical characteristics similar to that of the previous case and were treated by mechanical removal of the larvae, repeated washing and topical antibiotics. Multiple larvae were extracted from the conjunctiva of each patient and some of these larvae were sent for identification. However, due to poor state of preservation of the larvae that were severely damaged and fragmented, only molecular diagnostics allowed to identify extracted larvae as *O. ovis*. Genomic DNA was extracted with a commercial kit (NucleoSpin tissue kit, Macherey-Nagel, Duren, Germany), following the manufacturer’s instructions. As previously described (Otranto et al. 2003), the most variable part of the COI gene was amplified by PCR using two sets of conserved primers (UEA7–UEA8 and UEA8–UEA9) (Zhang and Hewitt 1997), which yielded a fragment of 688 bp. The PCR mix consisted of 3 μL of genomic DNA, 1× buffer including 1.5 mM MgCl_2_, 0.2 mM of each dNTP, 25 pM of each primer, and 1 unit of polymerase (BIOTAQ™ DNA Polymerase, Aurogene, Rome, Italy) in a final volume of 25 μL. PCR was performed as follows: after an initial denaturation step of 10 min at 94 °C, a set of 40 cycles was run, each consisting of 1 min at 94 °C, 1 min at 60 °C, 1 min at 72 °C, followed by a final extension of 7 min at 72 °C. PCR products were detected in a 2 % agarose TBE gel. The obtained amplicons were purified using the SureClean kit (Aurogene, Rome, Italy), following the manufacturer’s instructions and directly sequenced with PCR primers in both directions by an external sequencing core service (Eurofins MWG Operon, Anzinger, Germany). Sequences were corrected by visual analysis of the electropherograms, aligned using the MEGA v5.1 package (Tamura et al. 2011) and compared with the most similar sequences available from the National Centre for Biotechnology Information database (http://www.ncbi.nlm.nih.gov) using the BLAST program. Sequences analysis allowed the identification of the extracted larvae as *O. ovis* as yielded an identity score of 100 % with the *O.ovis*/specific COI partial gene (accession number AF497767).

### Review of literature of human myiasis in Italy

A review of PubMed indexed literature was performed using keywords “myiasis” and “Italy” in October 2013 without any language or period limit. Only articles reporting human cases of myiasis were included and one article (Pampiglione 1957a) was excluded because the full text was not available.

#### Autochthonous myiasis

Twenty-three papers concerning human myiases acquired in Italy, published in the period of 1957–2013, were found (Supplementary results and references). A total of 703 autochthonous cases of myiasis were reported, 690 (98.1 %) caused by *O. ovis.* In the middle of 1950s, 414 Italian doctors (8 % of interviewed) reported at least one case of *O. ovis* myiasis, 77 % of whom diagnosed in shepherds (Pampiglione 1958a). In the same period, 101 shepherds (85.6 % of interviewed) reported to be affected at least one time in their lives (Pampiglione 1958b). Most cases were observed from March to September, mostly in centre-southern Italy. Shepherds reported mainly oral, nasal, tonsillar and pharyngeal locations, whereas the conjunctival location was the most commonly observed by physicians, probably because the involvement of the eye prompts people to seek medical advice. Similar surveys carried out on shepherds (in the 1960s in Lazio region and in 1995 in Sicily) evidenced that 86.9 % of 73 interviewed and 90/112 (80.3 %), respectively, had been affected by myiasis at least one time in their lives (Pampiglione et al. 1997; Sacca et al. 1965). All surveys showed that shepherds are well aware of *O. ovis* myiasis, probably from centuries, and usually able to manage by themselves this problem by removing the maggots with the help of a friend or just waiting some weeks until the problems would be spontaneously solved. After 1995, seven sporadic cases of *O. ovis* have been reported in Italy, six of which in subjects without history of contacts with environment associated to sheep, goat or other cattle animals (Crotti et al. 2005; Dono et al. 2005; Otranto et al. 2009; Rivasi et al. 2009).

Only 13 cases of non-*O. ovis* autochthonous myiasis were reported in Italy, 10 of them (76.9 %) after the year 2000. Eight cases were caused by different species of flies belonging to Sarcophagidae family (that included *Sarcophaga* and *Wohlfahrtia* genera) (Billi et al. 1997; Dutto and Bertero 2010; Dutto and Bertero 2011; Dutto et al. 2012; Iori et al. 1999; Magliulo et al. 2000; Panu et al. 2000; Zardi et al. 2002), three caused by species of Calliphoridae family (that included *Lucilia* and *Calliphora* genera) (Dutto et al. 2010; Franza et al. 2006; Salvetti et al. 2011) and the remaining two cases were caused by *Hypoderma lineatum* (Pampiglione 1957b) and *Clogmia albipunctata* (Gelardi et al. 2009). All but one of cases due to Sarcophagidae and Calliphoridae families (10 cases) were wound myiasis in which the larvae developed in a preexistent lesion such as trauma wound (Dutto and Bertero 2010; Salvetti et al. 2011), surgical wound (Billi et al. 1997; Franza et al. 2006), chronic ulcers of lower limbs (Dutto et al. 2012; Dutto et al. 2010), neoplastic fistula (Zardi et al. 2002), eardrum perforated (Panu et al. 2000), preexistent dermatological condition (Iori et al. 1999) or macerated tissue in a skin fold (Dutto and Bertero 2011), whereas one case was an aural myiasis without previous ear damage (Magliulo et al. 2000). Eight of these cases were diagnosed in non self-sufficient, severely ill or geriatric patients. The case due to *H. lineatum*, an obligate parasite of ruminants, was described in a 13-year-old schoolboy with anterior internal ophthalmomyiasis (Pampiglione 1957b), while the case due to *C. albipunctata,* [=*Telmatoscopus albipunctatus,* Diptera*:* Psycodidae], a rarer agent of human wound and body cavities myiasis, was diagnosed in a 32-year-old women with nasal myiasis, working as an architect at the archaeological site of Pompeii (Gelardi et al. 2009). Patients with non-*O. ovis* autochthonous myiasis were treated by mechanical non-surgical extraction and surgery in nine and four cases, respectively.

#### Imported myiasis

Twenty-one papers reporting 42 imported cases, published in the period 1965–2012, were found (Supplemental results and supplemental references 25–45). All were furuncular myiases, 30 of them (71 %) reported from 2000 onwards. Twenty-five (59.5 %) had been acquired in Africa (17 from Senegal) and caused by *Cordylobia anthropophaga* (in 23 cases) and by *Cordylobia rodhaini* and *Cordylobia* spp. (one case each). About half (58.3 %) of the patients with *C. anthropophaga* acquired the infestation probably visiting African beaches. Seventeen cases (40.5 %) caused by *Dermatobia hominis* were imported from Latin America (five from Brazil). Furuncular-like lesions involved skin of limbs (primarily lower limbs for *Cordylobia* spp.), trunk and scalp (*D. hominis* only). Surgery to extract the larvae was more frequently applied in *D. hominis* cases (87.5 %) than in *Cordylobia* cases (53.8 %), as maggots of the Latin American species penetrate in tissues deeper than those of African species. Moreover, most cases (90 %) due to *Cordylobia* spp. were diagnosed within 7 days after the return, whereas the majority of those due to *D. hominis* (83 %) were diagnosed 2 weeks or more after return from endemic area, reflecting the different time required to the two species to develop in animal tissues. The number of furuncular lesions per patient was mostly one or two, but it reached 150 in an exceptional case of C. *rodhaini* infestation (Pampiglione et al. 1991).

Table 1 summarizes the characteristics of the principal causative agents of human myiasis reported in Italy.

**Table 1:** Characteristics of principal causative agents of human myiasis reported in Italy (Francesconi and Lupi 2012)

Fly species or family	Geographical distribution	Ecological classification and hosts	Clinical manifestations	Mode of infestation	Residence time in human tissues	Main treatment options in humans	Prognosis in humans
*Oestrus ovis*	Worldwide where sheep are bred. Mostly in Mediterranean basin including Italy and the Middle East	Obligatory parasite of sheep and goat	Ophthalmomyiasis externa; ENT myiasis	Adult females project their larvae on the face (or muzzle) of humans (or sheep) while flying	10–20 days (Macdonald et al. 1999; Pampiglione 1958a); probably not able to complete life cycle in humans	Mechanical removal; local anaesthetic to immobilize larvae and facilitate extraction. Few cases treated with ivermectin	Usually fair
*Dermatobia hominis*	Latin America	Obligatory parasite of livestock, humans and many other mammals	Furuncular myiasis (frequent localizations: scalp, face, extremities)	Eggs are laid on foliage or on vector insects^a^ (mosquitoes or other flies). Eggs hatch when entering in contact with a warm-blooded animals. Larvae penetrate the skin and start to develop	5–10 weeks	Occlusion of the breathing hole (with petroleum jelly or other staff) to force the larva to emerge and use of forceps when the larva became visible. Surgical incision if occlusion strategy fails	Usually fair. Bacterial superinfections possible
*Cordylobia anthropophaga*	Africa	Obligatory parasite of animals (mainly rats and dogs) and humans	Furuncular myiasis (frequent localizations: trunk, buttocks, thighs)	Eggs are laid on sandy ground or on clothes. Larvae penetrate the skin and start to develop	8–10 days	See *D. hominis* treatment option. Usually easier to remove compared with *D. hominis* (do not migrate in depth into tissues). Expression of furuncular lesion may be enough	Usually fair. Bacterial superinfections possible
Family Sarcophagidae	Depending on species	Obligatory parasite of humans and warm blooded animals	Wound myiasis (more rarely furuncular myiasis, or body cavity myiasis)	Larvae are directly deposited in wounds or other sites	7 days (Zumpt 1965)^b^	Mechanical or surgical removal of larvae with debridement of necrotic tissue	Depending on underling conditions of patient

*ENT* ear-nose-throat

^a^Adult females of *Dermatobia hominis* attach eggs to the abdomen of a bloodsucking intermediary mosquito or non-biting flies of the family Muscidae, which feed on liquid secretions such as sweat. This method of egg delivery is called phoresis

^b^This time may vary according to different species. Seven days is referred to *Wohlfahrtia magnifica*

## Discussion

We reported three cases of *O. ovis* conjunctival myiasis with a typical clinical pattern, one acquired in a public garden of the city centre of Florence, an unusual environmental scenario and two additional cases acquired in the Florence neighbouring areas. Previous publications report high frequency of conjunctival and otorhinolaryngological myiases by *O. ovis* in Italian shepherds, configuring myiasis in Italy mainly as an occupational disease up to the middle of 1990s, at least according to the published scientific literature (Pampiglione et al. 1997).

Following these years, only sporadic *O. ovis* human infestations have been reported, mainly in non-shepherds (Crotti et al. 2005; Dono et al. 2005; Mazzeo et al. 1987; Otranto et al. 2009), one of them acquired in urban setting (Otranto et al. 2009). The reduction in detected human cases has not been accompanied by the reduction of the burden of oestriasis in Italian sheep. In the matter of fact, some surveys still report prevalence in sheep of 73 % in 1989–1990 in Tuscany and 75 % in 2004 in Sardinia (Marconcini and Ercolani 1990; Suarez et al. 2005). The reduction of *O. ovis* cases in humans is probably related to the modernization of breeding techniques that led to a reduction of the prolonged contacts between humans and grazing lands that were a characteristic of Italian shepherds’ life until some decades ago (Pampiglione 1958b). A plausible explanation for the case acquired in the centre of Florence is the transport of an adult female fly from the countryside through a motor vehicle, like the case acquired in the Bari city centre, which was hypothesized as due to the transport of a female fly through a ship-transporting livestock from Greece or Albania (Otranto et al. 2009). Indeed, the occurrence in the same area of two additional cases of conjunctival myiasis caused by *O. ovis* suggests that the province of Florence could be a low endemic area for *O. ovis*. It is difficult to say if these three cases diagnosed in a 2-year period represent a real increase of incidence of human oestriasis in the Florence area. A more systematic reporting of human cases could be warranted together with veterinary studies to assess the current role of farm and wild animals as *O. ovis* reservoir in this area.

Human cases of *O. ovis* myiasis are becoming infrequent in Italy, whereas the review of literature shows that other myiases are increasingly diagnosed. The increasing number of international travels led to the emergence of furuncular myiasis cases, mainly after 2000. At the same time, reports of wound myiasis cases caused by flies belonging to Sarcophagidae and Calliphoridae families increased in the recent years, mainly in severely ill or geriatric patients, configuring myiasis in Italy also as an opportunistic, sometimes hospital-acquired, infection. The real magnitude of wound myiasis is likely to be underestimated because this kind of infestation is probably not considered so extraordinary and is likely unreported in the scientific literature. The etiologic agents of wound myiasis (including *Cochliomyia hominivorax*, *Chrysomya bezziana*, *Wohlfahrtia magnifica* and *Lucilia sericata*) are very common worldwide (Francesconi and Lupi 2012). The results of a multicentre prospective observational study carried out in the USA strongly support the underreporting of wound myiasis in scientific literature. In this study, 42 cases (71 % of which due to *L. sericata*) were diagnosed in a 3-year period (Sherman 2000). In the same paper, the authors presented a review of literature on human myiasis reported in USA in the period of 1960–1995 retrieving a relatively low number of cases (137) (Sherman 2000). The underreporting of wound myiasis is likely to occur in Italy as well. People who are in need of care (e.g. in a home environment with family or day care) and marginalized subjects such as homeless and unsheltered people are the most prone to develop this disease (Sherman 2000) and probably overlooked by the scientific literature.

Most myiases can be easily managed and have a good prognosis; however, the infestation is considered embarrassing and repugnant to patients and to health care professionals (Francesconi and Lupi 2012). Moreover, maggot infestations diagnosed in non self-sufficient patients may indicate inadequate care, may have significant psychological impact on patients and their families and can seriously harm the reputation of the involved healthcare facility (Sherman et al. 2005; Thyssen et al. 2012). Italian health care providers should know and implement the basic rules of entomoprophylaxis for myiasis in the facilities where they are working and educate patients, care giver and travellers accordingly (Table 2).

**Table 2:** Entomoprophylaxis measures recommended by Istituto Superiore di Sanità, Italy (Dutto et al. 2011)

Behavioural prophylaxis
Avoid activities in close contact with cattle (*Hypoderma* and *Oestrus*) Do not sleep outdoors near to livestock (particularly sheep) Take care to personal and environmental hygiene Cover any open wounds in exposed parts of the body Do not walk barefoot, especially on sandy soils (*Cordylobia*)
Chemical prophylaxis
Use repellent active against ticks and mosquitoes to prevent the phoretic transmission of *Dermatobia* ^a^
Prophylaxis physical
Use mosquito nets in the rooms where people are hospitalized with exposed/necrotic injuries (possibility of nosocomial myiasis) Ironing clothes (*Cordylobia*)
Mechanical prophylaxis
Use mosquito nets (*Dermatobia*)

^a^Adult females of *Dermatobia hominis* attach eggs to the abdomen of a bloodsucking intermediary mosquito or non-biting flies of the family *Muscidae*, which feed on liquid secretions such as sweat. This method of egg delivery is called phoresis

## Electronic supplementary material

Below is the link to the electronic supplementary material.

ESM 1 (DOC 139 kb)
