# Supplementary material for: Human oestriasis acquired in Florence and review on human myiasis in Italy
Source: Parasitol Res. 2014 May 7;113(6):2379–85. doi: 10.1007/s00436-014-3906-9 (PMC4031384; doi:10.1007/s00436-014-3906-9)
Supplement: Supplementary file 1 — (DOC 139 kb) [file 436_2014_3906_MOESM1_ESM.doc]

**Supplementary results and references**

**Journal:** Parasitology research

**Article title:** Human oestriasis acquired in Florence and review on human myiasis in Italy

**Authors:** Lorenzo Zammarchi^1^, Andrea Giorni^2^, Simona Gabrielli^3^, Marianne Strohmeyer^1^, Gabriella Cancrini^3^, Alessandro Bartoloni^1^.

**Affiliations**

1. Clinica Malattie Infettive, Dipartimento di Medicina Sperimentale e Clinica, Università Degli Studi di Firenze, Firenze, Italy

2. SOD Oculistica, Azienda Ospedaliera Universitaria Careggi, Firenze, Italy

3. Dipartimento di Sanità Pubblica e Malattie Infettive, Università “Sapienza”, Roma, Italy

The authors state that they have no conflict of interest

**Corresponding author:**

Alessandro Bartoloni

Largo Brambilla 3

50134 Florence

Italy

Email: [alessandro.bartoloni@unifi.it](mailto:alessandro.bartoloni@unifi.it)

Telephone and fax 0039-055-7949431

**Supplementary results**

Twenty three papers concerning human myiasis acquired in Italy were identified .

Twenty one papers concerning imported cases in Italy were identified .

**Supplementary references**

Billi B, et al. (1997) Pars plana vitrectomy for retinal detachment due to internal posterior ophthalmomyiasis after cataract extraction. Graefes Arch Clin Exp Ophthalmol 235(4):255-8

Bongiorno MR, Pistone G, Arico M (2007) Myiasis with *Dermatobia hominis* in a Sicilian traveller returning from Peru. Travel Med Infec Dis 5(3):196-8 doi:10.1016/j.tmaid.2006.09.007

Calderaro A, et al. (2008) Myiasis of the scalp due to *Dermatobia hominis* in a traveler returning from Brazil. Diagn Microbiol Infect Dis 60(4):417-8 doi:10.1016/j.diagmicrobio.2007.10.021

Crotti D, D'Annibale ML, Ricci A (2005) [A case of ophthalmomyiasis: description and diagnosis]. Infez Med 13(2):120-2

Cultrera R, Dettori G, Calderaro A, Iori A, Contini C (1993) [Cutaneous myiasis caused by *Cordylobia anthropophaga* (Blanchard, 1872): description of 5 cases froma coastal regions of Senegal]. Parassitologia 35(1-3):47-9

Dono M, et al. (2005) Three cases of ophthalmomyiasis externa by sheep botfly *Oestrus ovis* in Italy. New Microbiol 28(4):365-8

Dutto M, Bertero M (2010) Traumatic myiasis from *Sarcophaga* (Bercaeal cruentata Meigen, 1826 (Diptera, *Sarcophagidae*) in a hospital environment: reporting of a clinical case following polytrauma. J Prev Med Hyg 51(1):50-2

Dutto M, Bertero M (2011) Cutaneous superficial myiasis: report of a rare nosocomial parasitic disease caused by *Sarcophaga* spp. (diptera, sarcophagidae). Cent Eur J Public Health 19(4):232-4

Dutto M, Pellegrino M, Vanin S (2012) Nosocomial myiasis in a patient with diabetes. J Hospital Infect doi:10.1016/j.jhin.2012.08.019

Dutto M, Pomero F, Migliore E, Fenoglio L (2010) Cutaneous myiasis in a geriatric patient. Parassitologia 52(3-4):435-8

Franza R, Leo L, Minerva T, Sanapo F (2006) Myiasis of the tracheostomy wound: case report. Acta Otorhinolaryngol Ital 26(4):222-4

Fusco FM, Nardiello S, Brancaccio G, Rossiello R, Gaeta GB (2005) [Cutaneous myiasis from *Cordylobia anthropophaga* in a traveller returning from Senegal: a case study]. Infez Med 13(2):109-11

Gelardi M, Fiorella ML, Tarasco E, Passalacqua G, Porcelli F (2009) Blowing a nose black and blue. Lancet 373(9665):780 doi:10.1016/s0140-6736(09)60444-x

Guarneri C, Mughini M, Celesia B, Massimino D, Nunnari G (2012) The undesired fellow traveller. QJM 105(7):697-8 doi:10.1093/qjmed/hcr067

Guidi B, Olivetti G, Sbordoni G, Garcovich A (2001) Guess what! Diagnosis: cutaneous myiasis due to *dermatobia hominis*. Eur J Dermatol 11(3):259-60

Iori A, Zechini B, Cordier L, Luongo E, Pontuale G, Persichino S (1999) A case of myiasis in man due to *Wohlfahrtia magnifica* (Schiner) recorded near Rome. Parassitologia 41(4):583-5

Lodi A, Bruscagin C, Gianni C, Mancini LL, Crosti C (1994) Myiasis due to *Cordylobia anthropophaga* (Tumbu-fly). Int J Dermatol 33(2):127-8

Magliulo G, Gagliardi M, D'Amico R (2000) Human aural myiasis. Otolaryngol Head Neck Surg 122(5):777

Matera G, Liberto MC, Larussa F, Barreca GS, Foca A (2001) Human myiasis: an unusual imported infestation in Calabria, Italy. Journal of travel medicine 8(2):103-4

Mazzeo V, Ercolani D, Trombetti D, Todeschini R, Gaiba G (1987) External ophthalmomyiasis. Report of four cases. Int Ophthal 11(2):73-6

Novati S, Sacchi L, Chichino G, Scaglia M (1994) [Furuncular myiasis caused by *Cordylobia anthropophaga*: description of a case from Tanzania]. Parassitologia 36(3):265-7

Otranto D, Cantacessi C, Santantonio M, Rizzo G (2009) *Oestrus ovis* causing human ocular myiasis: from countryside to town centre. Clin Experiment Ophthalmol 37(3):327-8 doi:10.1111/j.1442-9071.2009.02026.x

Pampiglione S (1957) [Two further cases of ocular myiasis: anterior internal ophthalmomyiasis (fourth case in Italy) and conjunctival external ophthalmomyiasis]. Nuovi Ann Ig Microbiol 8(1):59-69

Pampiglione S (1958a) [Epidemiologic study of human conjunctival myiasis by *Oestrus ovis* in Italy. I. Survey among Italian doctors]. Nuovi Ann Ig Microbiol 9(3):242-63

Pampiglione S (1958b) [Epidemiological findings on human myiasis caused by *Oestrus ovis* in Italy. II. Survey on shepherds]. Nuovi Ann Ig Microbiol 9(6):494-517

Pampiglione S, Bettoli V, Cestari G, Staffa M (1993) Furuncular myiasis due to *Cordylobia anthropophaga*, endemic in the same locality for over 130 years. Ann Trop Med Parasitol 87(2):219-20

Pampiglione S, Giannetto S, Virga A (1997) Persistence of human myiasis by *Oestrus ovis* L. (Diptera: Oestridae) among shepherds of the Etnean area (Sicily) for over 150 years. Parassitologia 39(4):415-8

Pampiglione S, Schiavon S, Candiani G, Fioravanti ML (1991) [Clinical and parasitological observations on a case of disseminated furuncular myiasis caused by *Cordylobia rodhaini* in a man in Ethiopia]. Parassitologia 33(2-3):159-67

Panu F, Cabras G, Contini C, Onnis D (2000) Human auricolar myiasis caused by *Wohlfartia magnifica* (Schiner) (*Diptera*: *Sarcophagidae*): first case found in Sardinia. J Laryngology Otol 114(6):450-2

Pica R, Castellano C, Pignata D, Ipri D (2008) [Human cutaneous myiasis: a case report]. Clin Ter 159(6):431-3

Raffaldi I, Scolfaro C, Pinon M, Longo S, Savoia D, Tovo PA (2013) A strange gingival swelling in an Italian child: a case of oral myiasis. Infez Med 21(1):56-9

Rivasi F, Campi L, Cavallini GM, Pampiglione S (2009) External ophthalmomyiasis by *Oestrus ovis* larvae diagnosed in a Papanicolaou-stained conjunctival smear. Cytopathology 20(5):340-2 doi:10.1111/j.1365-2303.2008.00579.x

Rizzo G, De Vito D, Rizzo C (1998) [A case of cutaneous myiasis caused by *Dermatobia hominis*]. Parassitologia 40(3):335-7

Romano C, Albanese G, Gianni C (2004) Emerging imported parasitoses in Italy. Eur J Dermatol 14(1):58-60

Sacca G, Gabrielli L, Stella E (1965) [Notes on *Oestrus ovis* L. (*Diptera, Oestridae*) and description of some cases of myiasis in humans]. Ann Ist Super Sanita 1(1):73-94

Salvetti M, Corbellini C, Aggiusti C, Rosei EA, Muiesan ML (2011) *Calliphora vicina* human myiasis: a case report. Intern Emerg Med doi:10.1007/s11739-011-0720-6

Sperzani G (1965) [A case of external myiasis imported to Milan from Ghana (observation of particular interest for the route of infestation)]. Arch Ital Sci Med Parassitol 46(11):461-7

Urbani C, Simonacci M, Castelli P, Maroli M (1998) [Myasis due to *Cordylobia anthrophaga* (Diptera: Calliphoirdae): description of a clinical case and review of the literature]. Parassitologia 40(3):317-9

Varani S, et al. (2007) A case of furuncular myiasis associated with systemic inflammation. Parasitol Int 56(4):330-3 doi:10.1016/j.parint.2007.06.002

Veraldi S, Brusasco A, Suss L (1993) Cutaneous myiasis caused by larvae of *Cordylobia anthropophaga* (Blanchard). Int J Dermatol 32(3):184-7

Veraldi S, Francia C, Persico MC, La Vela V (2009) Cutaneous myiasis caused by *Dermatobia hominis* acquired in Jamaica. West Indian Med J 58(6):614-6

Veraldi S, Gorani A, Schianchi R (1998a) Guess What! Non-inflammatory cutaneous myiasis caused by the larva of *Cordylobia anthropophaga*. Eur J Dermatol 8(2):133-4

Veraldi S, Gorani A, Suss L, Tadini G (1998b) Cutaneous myiasis caused by *Dermatobia hominis*. Pediatr Dermatol 15(2):116-8

Zardi EM, Iori A, Picardi A, Costantino S, Petrarca V (2002) Myiasis of a perineal fistula. Parassitologia 44(3-4):201-2
